# Highly conserved and extremely evolvable: BMP signalling in secondary axis patterning of Cnidaria and Bilateria

**DOI:** 10.1007/s00427-024-00714-4

**Published:** 2024-03-13

**Authors:** David Mörsdorf, Paul Knabl, Grigory Genikhovich

**Affiliations:** 1 Dept. Neurosciences and Developmental Biology, University of Vienna, UBB, Djerassiplatz 1, 1030 Vienna, Austria; 2 Vienna Doctoral School of Ecology and Evolution (VDSEE), University of Vienna, Vienna, Austria

**Keywords:** BMP signalling, Bilateria, Bone morphogenetic protein

## Abstract

Bilateria encompass the vast majority of the animal phyla. As the name states, they are bilaterally symmetric, that is with a morphologically clear main body axis connecting their anterior and posterior ends, a second axis running between their dorsal and ventral surfaces, and with a left side being roughly a mirror image of their right side. Bone morphogenetic protein (BMP) signalling has widely conserved functions in the formation and patterning of the second, dorso-ventral (DV) body axis, albeit to different extents in different bilaterian species. Whilst initial findings in the fruit fly *Drosophila* and the frog *Xenopus* highlighted similarities amongst these evolutionarily very distant species, more recent analyses featuring other models revealed considerable diversity in the mechanisms underlying dorsoventral patterning. In fact, as phylogenetic sampling becomes broader, we find that this axis patterning system is so evolvable that even its core components can be deployed differently or lost in different model organisms. In this review, we will try to highlight the diversity of ways by which BMP signalling controls bilaterality in different animals, some of which do not belong to Bilateria. Future research combining functional analyses and modelling is bound to give us some understanding as to where the limits to the extent of the evolvability of BMP-dependent axial patterning may lie.

## Brief overview of the BMP signalling pathway

**Fig. 1:**
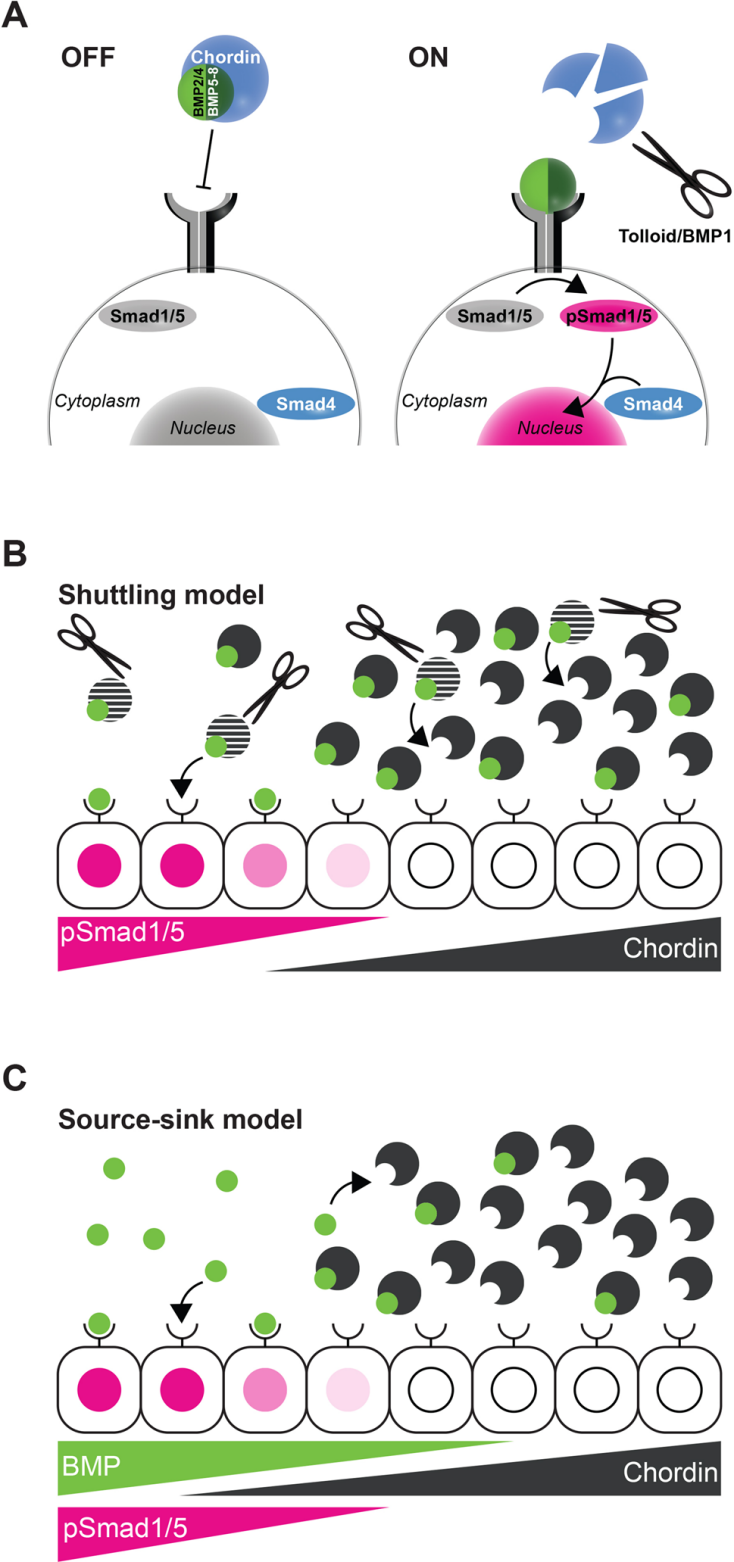
BMP signalling. **A** Illustration of the core BMP signalling components. BMP dimers are bound and inhibited by Chordin, which blocks them from interacting with the BMP receptor complex (left). In the absence of Chordin, or after Tolloid-catalysed cleavage of Chordin and release of the BMP ligand, BMPs activate the receptor complex leading to phosphorylation of Smad1/5 (indicated by pink colour). Phosphorylated Smad1/5, together with the Co-Smad Smad4, enters the nucleus and regulates transcription of BMP target genes (right). **B** BMP-mediated patterning according to the shuttling model: close to the Chordin source, a high concentration of Chordin blocks BMPs from binding their receptors. The BMP-Chordin complex is diffusible and can effectively transport BMPs. Only at a distance of the Chordin source, release of BMPs from the inhibitory complex after Tolloid cleavage enables BMPs to bind receptors rather than another, yet uncleaved, Chordin molecule. In the shuttling model, BMP signalling is activated at a distance from the Chordin source, independent of the BMP source location. BMP protein concentration profile deliberately not shown since in the shuttling model it is irrelevant for the location of the BMP signalling domain (see Genikhovich et al. 2015). **C** BMP signalling in the source-sink model: BMP and Chordin source oppose each other. BMPs form a concentration gradient from their source and activate receptor complexes where they can bind them. Chordin forms an opposing gradient and BMP signalling is inhibited where Chordin concentration is high. Chordin acts locally to inhibit BMP signalling and does neither to inhibit, nor promote BMP signalling at a distance. Pink colour indicates phosphorylated Smad1/5 (active BMP signalling) in all panels

BMPs are secreted signalling proteins and members of the transforming growth factor-β (TGF-β) superfamily. The TGF-β signalling pathway is one of the evolutionary novelties of animals (Babonis and Martindale 2017; Huminiecki et al. 2009). Mature BMP ligands are produced from pro-proteins by proteolytic processing and act as homo- or heterodimers, with heterodimers tending to be the “stronger” signals (Aono et al. 1995; Bauer et al. 2023; Cui et al. 2001; Fritsch et al. 2012; Hazama et al. 1995; Künnapuu et al. 2009, 2014; Little and Mullins 2009; Nishimatsu and Thomsen 1998; Shimmi et al. 2005; Sopory et al. 2010; Suzuki et al. 1997; Tajer et al. 2021). The “main” BMP ligands belong to the BMP2/4 and BMP5-8 families, however, other BMPs, e.g. ADMP and GDF5-6, exist and play a role in DV patterning (Genikhovich et al. 2015; Lapraz et al. 2015; Reversade and De Robertis 2005). BMP dimers bind and activate heterotetrameric BMP receptor complexes, consisting of two type I and two type II receptors (reviewed in Heldin and Moustakas (2016)). Receptor complex activation triggers the phosphorylation of the receptor-regulated Smads (R-Smads), Smad1/5^1^ (Mad in *Drosophila*^2^) - transcriptional effectors that form a heterotrimeric complex with a so-called common-partner Smad (Co-Smad), Smad4 (Medea in *Drosophila*) and translocate into the nucleus, where they activate or repress gene expression assisted by a range of co-factors (reviewed in Hill (2016); Fig. 1A). Inhibition of BMP signalling is possible at different steps along the signalling pathway. For example, inside the cell, the R-Smad/Co-Smad complex action is counteracted by inhibitory Smads (I-Smads), Smad6 and Smad7, which inhibit R-Smad phosphorylation by interacting with the type I receptor, as well as by several other mechanisms (Miyazawa and Miyazono 2017), whilst at the cell surface, a non-functional type I BMP receptor BAMBI (BMP and Activin membrane-bound inhibitor) interferes with the assembly of active TGF-β receptor complexes (Onichtchouk et al. 1999). However, the already quite complex intracellular regulation of BMP signalling is easily eclipsed by the mind-boggling complexity of the BMP signalling regulation by extracellular proteins. Chordin, Noggin, Follistatin, Gremlin and Cerberus are extracellular BMP signalling inhibitors whose function is at least partially redundant (Bachiller et al. 2000; Brazil et al. 2015; Dal-Pra et al. 2006; Francois et al. 1994; Genikhovich et al. 2015; Holley et al. 1996; Iemura et al. 1998; Miller et al. 2019; Piccolo et al. 1999; Sasai et al. 1994; Tajer et al. 2021; Zimmerman et al. 1996). Amongst them, Chordin (Short gastrulation, Sog in *Drosophila*) stands out as a molecule with a dual anti-BMP and pro-BMP function. Similar to other inhibitors, Chordin binds BMP dimers preventing them from binding the receptors. On the other hand, Chordin-BMP complexes are capable of diffusion and, upon cleavage of Chordin by the metalloproteases Tolloid or BMP1, release BMP dimers promoting signalling at a distance from the Chordin source (Marqués et al. 1997; Piccolo et al. 1997; Scott et al. 1999; Shimmi et al. 2005; Tuazon et al. 2020; Wang and Ferguson 2005). Many other extracellular regulators of BMP signalling are known, especially in chordates: twisted gastrulation (Tsg) with its complex pro- and anti-BMP activities, a Tolloid antagonist Sizzled, a Tolloid agonist Ont1, another Tolloid inhibitor Crescent, an ADMP regulator Pinhead, and more (Chang et al. 2001; Dal-Pra et al. 2006; Imai et al. 2012; Inomata et al. 2008; Itoh et al. 2021; Lee et al. 2006; Oelgeschlager et al. 2000; Peluso et al. 2011; Ploper et al. 2011; Ross et al. 2001; Scott et al. 2001; Shimmi and O'Connor 2003; Wang and Ferguson 2005; Yan et al. 2019).

## Diverse mechanisms establish BMP signalling gradients along the DV axis

BMPs in the *Drosophila* embryo were amongst the first morphogens to be discovered (Ferguson and Anderson 1992a). The two BMP ligands responsible for the formation of the BMP signalling gradient along the second body axis are the dorsally produced BMP2/4 (Decapentaplegic, Dpp) and the ubiquitously produced BMP5-8 (Screw) (Arora et al. 1994; Jazwinska et al. 1999; Shimmi et al. 2005). BMP dimers form a BMP-Chordin-Tsg complex diffusing in the perivitelline space. BMP is released from this inhibitory complex by Tolloid-mediated cleavage of Chordin, allowing BMP to bind its receptors (Eldar et al. 2002; Marqués et al. 1997; Peluso et al. 2011; Ross et al. 2001; Shimmi and O'Connor 2003; Shimmi et al. 2005; Wang and Ferguson 2005). A Chordin-mediated flux of BMPs, BMP-dependent Chordin cleavage by Tolloid, and feedback regulation by BMP target genes together generate a steep dorsal-to-ventral gradient of nuclear pSmad1/5 (Ashe and Levine 1999; Eldar et al. 2002; Gavin-Smyth et al. 2013; Mizutani et al. 2005; Peluso et al. 2011; Srinivasan et al. 2002; Wang and Ferguson 2005). The mechanism of BMP gradient formation, which requires Chordin-assisted BMP transport away from the Chordin source and does not depend on the location of the BMP source, has been termed “BMP shuttling” (Fig. 1B). Experiments in frog and sea urchin embryos support a similar model (Ben-Zvi et al. 2008; Lapraz et al. 2009, 2015; Plouhinec et al. 2013).

However, shuttling and the pro-BMP function of Chordin is not conserved in all animals. In zebrafish, for example, loss of Chordin function does not reduce the maximum in the BMP signalling gradient (Pomreinke et al. 2017; Zinski et al. 2017). Instead of promoting the extracellular dispersion of BMP ligands and BMP signalling at a distance to the Chordin source, zebrafish Chordin appears to only repress BMP signalling and act as BMP sink in a “source-sink” mechanism. This was elegantly shown by using membrane-tethered Chordin: localized expression of this immobile Chordin can rescue *chordin* mutants, if the expression resembles the endogenous expression domain (Tuazon et al. 2020; Fig. 1C). There are also bilaterian clades in which Chordin was lost, and which use other means to pattern their DV axis, as we will describe below.

## An overview of the BMP-dependent DV patterning across Bilateria

**Fig. 2:**
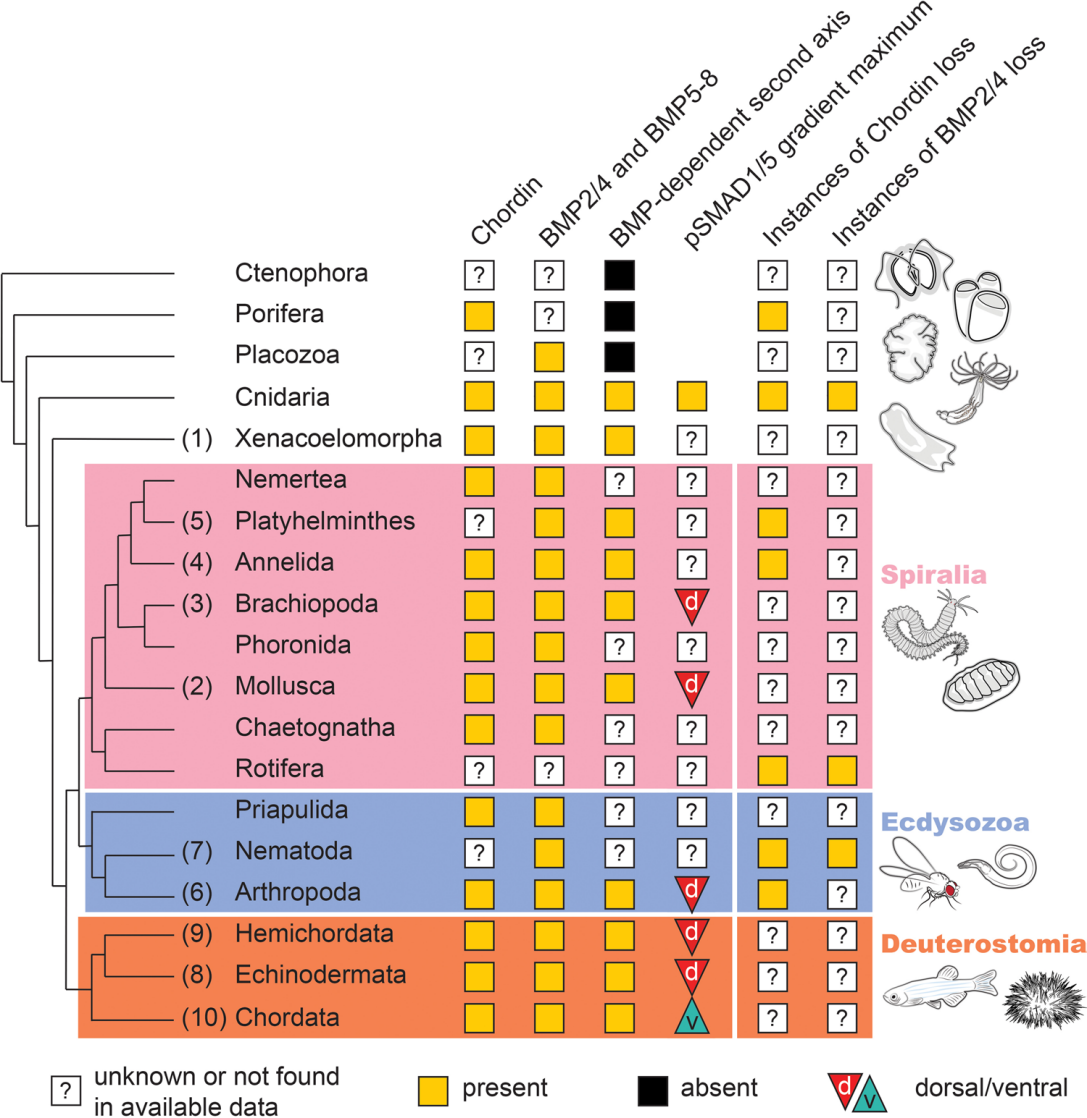
Involvement of BMP signalling in second body axis patterning in various phyla. Numbers at tips of the branches correspond to numbers of the sections in the text

Now, after a brief summary of the main BMP signalling components and their modes of action underlying BMP-mediated patterning, let us have a look at the implementation of the BMP signalling for DV patterning in Bilateria (Fig. 2; the numbers at the branches correspond to the numbers in the text).

### *(1) Xenacoelomorpha*

The phylogenetic position of Xenacoelomorpha is heavily disputed: currently, they are placed either as the earliest branching Bilateria, *i.e.* the sister group to “Nephrozoa”—a clade uniting deuterostomes and protostomes (Cannon et al. 2016), as a sister group to Ambulacraria (echinoderms and hemichordates) within Deuterostomia (Mulhair et al. 2022), or even as a sister group to Ambulacraria in a scenario where deuterostomes are considered paraphyletic (Kapli et al. 2021). In this paper, we will follow the views of Cannon et al. (2016); however, it is important to remember that the situation is far from resolved. Limited information on Xenacoelomorpha body axis patterning is available from studies of acoels. There is a complement of the main BMP signalling components including Chordin and three BMP ligands in *Hofstenia miamia*, including *Hof-bmp5-8*, a dorsally expressed *Hof-bmp* and a ventrally expressed *Hof-admp*.^3^ However, the phylogenetic relationship of Hof-BMP to the BMP ligands of other animals is not entirely clear, and it is unlikely that Hof-BMP is a BMP2/4 orthologue. RNAi has been used to deplete two BMP ligands (*Hof-bmp* and *Hof-admp*) and Smads (*Hof-smad1/5* and *Hof-smad4*) during regeneration, each condition leading to ventralisation (Srivastava et al. 2014). Moreover, *bmp2/4* is expressed dorsally in the acoel *Convolutriloba longifissura* (Hejnol and Martindale 2008). Together, these findings indicate that BMP signalling in acoels is required for the formation of dorsal structures. The role of acoel Chordin is yet unknown.

### *Protostomia/Spiralia*

Studies on various annelid, mollusc, brachiopod and planarian flatworm species are beginning to shed light on the function of BMP signalling in the early development of Lophotrochozoa/Spiralia. Although Chordin was lost in such established annelid models as the ragworm *Platynereis* and leech *Helobdella*, it is present in the early-branching annelid *Owenia fusiformis*, where an asymmetric expression along the dorsoventral axis has been reported (Martin-Zamora et al. 2023). The fact that BMP and Chordin are also implicated in DV patterning in brachiopods (Martin-Duran et al. 2016) and molluscs (Tan et al. 2022) indicates that BMP/Chordin-mediated dorsoventral patterning is an ancestral feature in Spiralia. However, DV patterning in Spiralia can be used as a perfect example of the extent of the developmental systems drift.

#### (2) Mollusca

In molluscs, BMP signalling was shown to have functions in secondary axis patterning in several cases, such as snails *Ilyanassa* (Lambert et al. 2016) and *Lottia goshimai* (Tan et al. 2022) where BMP2/4 depletion leads to at least a transient radialisation. The importance of Chordin in regulating DV patterning was demonstrated in *Lottia*, where asymmetric *chordin* expression is downstream of MAP kinase signalling from the organizer and required for asymmetric BMP signalling (Tan et al. 2022). Gene expression studies and BMP signalling inhibition indicate that BMP signalling similarly functions in DV axis formation in the oyster *Crassostrea gigas* (Tan et al. 2017, 2018). However, diversity in dorsoventral patterning exists also in molluscs; for example in the slipper snail *Crepidula fornicata*, BMP signalling was suggested not to impact DV patterning (Lyons et al. 2020).

#### (3) Brachiopoda

A study on axis formation in two brachiopod species, *Novocrania anomala* and *Terebratalia transversa*, reports that BMP signalling is active in the dorsal ectoderm, opposing the ventral *chordin* expression domain (Martin-Duran et al. 2016). Moreover, inhibition of BMP signalling results in ventralisation, but also anteriorization, which shows BMP-dependent secondary axis patterning in brachiopods and indicates that there is crosstalk between the dorsoventral and anterior–posterior patterning systems.

#### (4) Annelida

It was suggested that dorsoventral patterning could be fundamentally different between annelids and molluscs, since establishing the dorsoventral axis appears to be independent of BMP signalling in some annelid species such as *Capitella teleta*, where Activin/Nodal plays the central role in the DV patterning (Lanza and Seaver 2020; Webster et al. 2021). However, other annelids do use BMP signalling for DV patterning. The effect of the BMP signalling modulation in one of the most developed annelid models, *Platynereis*, suggests that dorsally expressed *BMP2/4* is involved in DV patterning of the neurectoderm, and that the genes responding to this BMP modulation are the same in *Platynereis*, *Drosophila* and vertebrates (Denes et al. 2007). In the future, it will be interesting to find out in detail how *Platynereis* DV patterning is regulated, since *Platynereis*, like *Capitella*, does not have Chordin. In some cases, annelid BMP-dependent DV patterning can be quite bizarre. In the leech *Helobdella*, it acts in a curious cell-to-cell relay way. The DV patterning takes place within four pairs of “germinal bandlets” called n, o, p, and q, where n is the ventralmost and q is the dorsalmost bandlet. Two *BMP2/4* and one *ADMP* gene are broadly expressed in all the bandlets, whilst BMP5-8 is only expressed in the dorsalmost q-bandlet and is the key BMP in this patterning process. BMP5-8 is necessary and sufficient to activate the expression of the BMP inhibitor Gremlin in the p-bandlet, which, in turn, is required for the specification of the o-bandlet, which is achieved by Gremlin apparently inhibiting BMP2/4-mediated but not BMP5-8-mediated signalling (Kuo and Weisblat 2011). In this context, it is important to repeat that *chordin* may be absent from many annelid genomes (including *Platynereis* and *Capitella*), but not from all. As already mentioned, the BMP-Chordin system appears to be involved in the DV patterning in the early-branching annelid *Owenia* (Martin-Zamora et al. 2023).

#### (5) Platyhelminthes

The function of BMP signalling in planarian flatworms has been studied mainly during regeneration of *Dugesia japonica* and *Schmidtea mediterranea*. Whilst it still remains to be shown by pSmad1/5 immunofluorescence where BMP signalling is active in the planarian body, RNAi of Smads (Smad1 and Smad4; Gavino and Reddien 2011; Reddien et al. 2007) indicates that it is required for the formation of dorsal structures. Consistent with this, RNAi of BMPs leads to ventralisation (Clark and Petersen 2023; Molina et al. 2007; Orii and Watanabe 2007; Reddien et al. 2007). Like in many other models, ventrally expressed ADMP acts in a feedback circuit with dorsal BMP4, probably to balance BMP signalling against fluctuations (Gavino and Reddien 2011; Molina et al. 2011). Intriguingly, a *chordin* gene has not been identified in planarians; however, Noggin and—unexpectedly, given the lack of Chordin—Tolloid homologs have been proposed to regulate BMP signalling in *Schmidtea* (Gavino and Reddien 2011; Molina et al. 2011; Reddien et al. 2007). In contrast, the function of *noggin-like* genes in *Dugesia* (Ogawa et al. 2002; Orii and Watanabe 2007) is not clear. Further research is necessary to understand the mechanism of the BMP-dependent DV patterning in flatworms, but this is hampered by the lack of a developed embryonic model.

### *Protostomia/Ecdysozoa*

#### (6) Arthropoda

Work in different arthropods has provided fundamental insights into the functions of BMP signalling during development and the evolution of BMP-mediated patterning. Experiments in *Drosophila melanogaster* have been instrumental in our understanding of the BMP signalling pathway, from heterodimer formation and secretion (Bauer et al. 2023; Shimmi et al. 2005), to interactions with extracellular regulators (Peluso et al. 2011; Shimmi and O'Connor 2003), to extracellular transport and signalling (Simon et al. 2023), to the regulation of BMP target genes (Hoppe et al. 2020; Wharton et al. 2004). Nowadays, it is even possible to characterize the kinetics along the different steps of the signalling pathway (Romanova-Michaelides et al. 2022). Whilst the mechanisms underlying dorsoventral patterning discovered in *Drosophila* have been largely accepted as universal and even transferred to other model systems (BMP shuttling in frog, sea urchin), it is becoming clear that even within Arthropoda they are much more variable than initially expected.

BMP signalling is the strongest in the dorsal-most cells in the early *Drosophila* embryo (Mizutani et al. 2005; Peluso et al. 2011; Ross et al. 2001; Shimmi and O'Connor 2003). As we described above, the narrow domain of peak BMP signalling activity is explained by a shuttling mechanism that concentrates BMP ligands in the target domain located on the side of the embryo opposite to the Chordin source and is supported by transcription-based positive feedback (Eldar et al. 2002; Gavin-Smyth et al. 2013; Wang and Ferguson 2005). The detailed examination of DV patterning in *Drosophila* has revealed the intricate mechanisms regulating shuttling which involve not only Chordin and Tolloid, but also Tsg. Tsg can form a ternary complex with BMP and Chordin that efficiently inhibits BMP signalling and modulates Chordin cleavage (Eldar et al. 2002; Marqués et al. 1997; Peluso et al. 2011; Ross et al. 2001; Shimmi and O'Connor 2003; Shimmi et al. 2005; Wang and Ferguson 2005). Disruption of any of the shuttling components leads to DV patterning defects. Interestingly, *Drosophila* mutants for genes encoding BMP ligands are ventralised but do not display complete radialisation of the dorsoventral axis (Arora et al. 1994; Ferguson and Anderson 1992a, b). This is due to the determination of DV polarity upstream of BMP signalling in insects, where Toll/Dorsal signalling determines ventral cell fates (reviewed in Roth 2023).

However, flies are one of the younger insect clades. Moving from *Drosophila* towards insects in earlier branching groups has shown the decreasing importance of Toll signalling and the increasing role of BMP signalling in controlling the DV patterning in the flour beetle *Tribolium castaneum* (Nunes da Fonseca et al. 2008; van der Zee et al. 2006), the parasitoid wasp *Nasonia vitripennis* (Özüak et al. 2014) and the milkweed bug *Oncopeltus fasciatus* (Sachs et al. 2015). In *Tribolium*, pSmad1/5 is detected on the dorsal side of the embryo and requires the BMP2/4, Tc-dpp. This dorsal BMP signalling extends beyond the *Tc-dpp* expression domain and the dorsal restriction of signalling requires ventrally-expressed Chordin. This indicates that a shuttling-like system is in place (van der Zee et al. 2006). But, similar to *Drosophila*, Toll signalling is required in *Tribolium* to establish the DV axis (Nunes da Fonseca et al. 2008). In the wasp *Nasonia*, the influence of Toll signalling on DV patterning is reduced as embryos depleted of Toll retain DV polarity. Moreover, BMP2/4 (Nv-dpp) knockdown ventralises not only dorsal but also lateral tissue, revealing a stronger influence of BMP signalling on DV patterning than in *Drosophila*. Interestingly, DV patterning in the wasp appears to be independent of a Chordin, as no homolog was identified in *Nasonia* (Özüak et al. 2014). In the milkweed bug *Oncopeltus*, Chordin and Tsg are important for restricting BMP signalling to dorsal cells, allowing the formation of mesoderm at the opposing, ventral side. Chordin expression is negatively regulated by BMP signalling, as depletion of BMP2/4 or Tld ventralises the embryo and expands the *chordin* expression domain. Whilst Toll1 knockdown leads to a complete dorsalisation of the *Oncopeltus* embryo, knockdown of Chordin or BMP also abolishes DV patterning. It was proposed that Toll signalling is only required to provide initial DV asymmetry in *Oncopeltus* and that BMP signalling regulates the major part of DV patterning (Sachs et al. 2015). Counterintuitively, the DV patterning in the cricket *Gryllus bimaculatus*, which belongs to an earlier branching arthropod group than bugs, wasps and beetles, is very similar to *Drosophila* (Pechmann et al. 2021). Here, Toll is required to polarize the dorsoventral axis and Toll1 knockdown leads to the loss of ventral and lateral tissues. In contrast, depletion of BMP signalling components only abolishes dorsal structures and does not interfere with the mesoderm specification. Moreover, Chordin, which is crucial for dorsoventral patterning in *Drosophila*, is not present in *Gryllus* and other *Gryllidae*. However, given that in non-insect arthropods, such as the spider *Achaearanea tepidariorum*, both Chordin and BMP2/4 are required for proper dorsoventral patterning, it is clear that the cricket/fly similarity resulted from the independent loss of the leading role of BMP signalling in the DV patterning rather than represents an ancestral arthropod condition. In the spider embryo, *chordin* RNAi results in expanded BMP signalling and loss of ventral structures, whilst depletion of *bmp2/4* transcripts leads to a reduction in dorsal tissues with an almost complete radialisation (Akiyama-Oda and Oda 2006).

Taken together, BMP-Chordin-based DV pattering was clearly ancestral for Arthropoda; however, this highly diverse animal group makes a prime example of how a patterning system can be independently modified and adjusted multiple times during evolution.

#### (7) Nematoda

In the nematode *Caenorhabditis elegans*, BMP signalling pathway components are present; however, they do not regulate embryonic dorsoventral patterning (Patterson and Padgett 2000). Nonetheless, they do have functions in later development and, potentially, patterning. For example, the BMP-like DBL-1 is important to regulate body size, and repression of BMP signalling involving DBL-1 is required for the dorsoventral patterning of the postembryonic mesoderm (Shen et al. 2018; Suzuki et al. 1999). Interestingly, although Chordin is lost in advanced nematodes, it was found in early branching nematodes *Romanomermis culicivorax* and *Trichinella spiralis* as well as in a member of a closely related ecdysozoan phylum Nematomorpha, *Gordius sp*. (Kraus 2015). However, the function of Chordin and BMP signalling in general in these animals remains unknown.

### *Deuterostomia*

#### (8) Echinodermata

Although pentaradially symmetric as adults, echinoderm larvae are bilaterally symmetric and have a BMP-dependent DV axis, whose establishment and patterning have been analysed in sea urchin embryos. Unusually, the sea urchin gene with the earliest known bilaterally symmetric expression is *Nodal*, which is activated on the future ventral side of the *Paracentrotus lividus* blastula and activates the expression of *bmp2/4* and *admp1*, but also *chordin* (Chang et al. 2016; Duboc et al. 2004; Lapraz et al. 2009, 2015). Whilst elegant experiments have shown that Chordin is required to shuttle BMP ligands and promote BMP signalling on the dorsal side, BMP itself (in the absence of Chordin) can phosphorylate Smad1/5 throughout the embryo, albeit only to a low level (Lapraz et al. 2009, 2015). ADMP2 is a dorsally-expressed ligand that additionally promotes dorsal pSmad1/5 and is under positive control of BMP signalling (Chang et al. 2016; Lapraz et al. 2015).

#### (9) Hemichordata

DV axis patterning was examined in two acorn worm species, the equally cleaving direct developer *Saccoglossus kowalevskii* (Lowe et al. 2006) and the unequally cleaving indirect developer *Ptychodera flava* (Su et al. 2019). In both cases, BMP signalling is required for secondary axis patterning. The BMP ligands (BMP2/4 and, in the case of *Saccoglossus*, also BMP5-8) are expressed dorsally, whereas *Chordin* is expressed on the ventral side (Lowe et al. 2006; Röttinger et al. 2015; Röttinger and Martindale 2011; Su et al. 2019). Treating embryos with recombinant zebrafish BMP4 leads to dorsalisation, whilst inhibiting BMP signalling pharmacologically or by BMP2/4 knockdown results in ventralisation (Lowe et al. 2006; Röttinger et al. 2015; Su et al. 2019). Also ADMPs are found in hemichordates: *ADMP2* is expressed dorsally in *Ptychodera* (Chang et al. 2016; Röttinger et al. 2015), whereas *ADMP1*—the orthologue of the *Saccoglossus ADMP* (Lowe et al. 2006)—is under negative control of BMP signalling and expressed ventrally together with *Chordin*. Some fragments of the gene regulatory network responsible for the hemichordate DV patterning are clearly still unknown, especially the role of Nodal, which, like in the sea urchin, is expressed on the ventral side of the *Ptychodera* embryo. Surprisingly, treating the embryos with recombinant mouse Nodal resulted in the same dorsalising effect as the treatment with recombinant zebrafish BMP4 (Röttinger et al. 2015), suggesting the Nodal signalling might activate the expression of a ventrally expressed BMP (maybe it is ADMP in *Saccoglossus*) and its shuttling by Chordin.

#### (10) Chordata

In the early gastrula of the lancelet *Branchiostoma*, a member of the **cephalochordates**, BMP2/4 and BMP5-8 are expressed vegetally without DV asymmetry, whereas Chordin and ADMP are expressed dorsally. This situation changes at later gastrula stages when BMP2/4 and BMP5-8 expression becomes suppressed dorsally (Yu et al. 2007). Perturbation of BMP signalling has shown that its inhibition leads to dorsalisation and activation of BMP signalling leads to ventralisation (Le Petillon et al. 2017; Onai et al. 2010; Yu et al. 2007). Already in cephalochordates we see the persistent chordate theme of the opposing activities of the dorsalising Nodal and the ventralising BMP (Onai et al. 2010). BMP signalling is also important for the downstream left–right asymmetry (Soukup and Kozmik 2018).

Work on sea squirts, which is a common name for ascidians—the members of the vertebrate sister group **Tunicata**, showed that BMP signalling mediated by the dorso-laterally expressed *ADMP*, and the ventrally expressed *BMP2/4* (and possibly also *BMP5-8*) is active in the ventral epidermis from late gastrula stages on (Imai et al. 2012; Roure et al. 2023; Waki et al. 2015). Chordin is expressed in two dorso-lateral stripes (Abitua et al. 2015) suggesting that it might be involved in antagonizing BMP activity on the dorsal side of the embryo. BMP signalling is required for the ectodermal DV patterning (Imai et al. 2012; Roure et al. 2023; Waki et al. 2015). ADMP-mediated BMP signalling and Chordin are also involved in the formation of the three palps on the head of the tadpole, which are specified in the pSmad1/5-negative domain at the so-called anterior neural border but subsequently require BMP signalling for their proper patterning (Darras and Nishida 2001; Roure et al. 2023; Liu et al. 2023). It has been suggested that ascidian BMP signalling has an “anti-neural” function during neural induction, since pharmacological inhibition of BMP signalling de-represses the dorsally expressed pro-neural transcription factor *Otx* (Ohta and Satou 2013). However, this statement, in our opinion, has to be amended. Similar to the situation in cephalochordates, BMP signalling in tunicates cannot be considered strictly anti-neural, as it is required for the specification of the ventral sensory neurons in both these lineages (Waki et al. 2015; Lu et al. 2012).

Finally, we come to the situation in **vertebrates**. Over the last 30 years, the **zebrafish** *Danio rerio* has become a powerful model system to investigate the functions of BMP signalling during vertebrate development. BMP signalling activity has been shown by pSmad1/5 immunofluorescence to be restricted to the ventral side during early gastrulation (Greenfeld et al. 2021; Pomreinke et al. 2017; Ramel and Hill 2013; Rogers et al. 2020; Tucker et al. 2008; Zinski et al. 2017). The expression of BMP ligands BMP4 and BMP2b is restricted to ventral tissues and, curiously, the dorsal organizer, during early gastrulation (Dick et al. 2000; Kishimoto et al. 1997; Ramel and Hill 2013; Schmid et al. 2000; Xue et al. 2014). Mutants for the BMP ligands BMP2b and BMP7a lack ventral tissues, such as the tail and blood (Dick et al. 2000; Mullins et al. 1996; Schmid et al. 2000). Chordin is expressed dorsally in the organizer (Hammerschmidt et al. 1996; Kishimoto et al. 1997; Miller-Bertoglio et al. 1997). Loss of Chordin function leads to dorsal expansion of the pSmad1/5 domain and dorsal expansion of ventral marker genes (Hammerschmidt et al. 1996; Pomreinke et al. 2017; Zinski et al. 2017). The mechanisms underlying BMP signalling-mediated patterning have been intensively studied over the last years. Amongst the tested models was also the shuttling of BMP ligands by Chordin, which we discussed above. In zebrafish, however, Chordin does not exhibit any pro-BMP functions and merely seems to dampen BMP signalling, as evident from *chordin* mutants that have an expanded, but not flattened pSmad1/5 gradient (Pomreinke et al. 2017; Zinski et al. 2017). Moreover, extracellular dispersion of Chordin, which is crucial for Chordin function in the shuttling model (Ashe and Levine 1999), is not required for its function in zebrafish, as shown by experiments using a membrane-tethered Chordin (Tuazon et al. 2020). We have to note that the dorsally-expressed ADMP also functions in dorsoventral patterning (Dickmeis et al. 2001; Lele et al. 2001; Yan et al. 2019), and it can currently not be excluded that Chordin shuttles this BMP ligand as in frogs (Pomreinke et al. 2017; Reversade and De Robertis 2005; Reversade et al. 2005). BMPs are usually considered textbook examples of morphogens acting to pattern the DV axis (Bier and De Robertis 2015). However, the quantification of the pSmad1/5 gradient and BMP target gene expression in zebrafish has revealed that the classical threshold-dependent target gene activation is true only to a certain extent, and that crosstalk between signalling pathways is critical to pattern complex tissues (Greenfeld et al. 2021; Rogers et al. 2020).

Analysis of the DV patterning in amphibians started somewhat gloriously with the discovery of the dorsalizing, axis-inducing activity conveyed by the Spemann organizer (Spemann and Mangold 1924). *Chordin* was first identified as an organizer gene with the potency to induce axis duplication upon misexpression in the **frog** *Xenopus* (Sasai et al. 1994). Chordin binds the ventrally-expressed BMP4 as well as BMP4-BMP7 heterodimers, and blocks the BMP ligand from binding to its receptors (Dale et al. 1992; Piccolo et al. 1996). BMP4 itself ventralises embryos and represses dorsal organizer genes like *goosecoid* (Fainsod et al. 1994; Jones et al. 1996). The proper formation of ventral structures requires in addition to BMP4 the dorsally-produced ADMP, as well as BMP2 and BMP7, which are expressed along the whole DV axis (Hawley et al. 1995; Hemmati-Brivanlou and Thomsen 1995). Only a combined loss of BMP2, BMP4, BMP7 and ADMP leads to a radialisation of the frog embryo (Reversade and De Robertis 2005). Although the general components of dorsoventral patterning and their expression domains are very similar to those in zebrafish embryos, a more active function of Chordin in the patterning of *Xenopus* was proposed. There is evidence that Chordin does not only act as a dorsal inhibitor of BMP signalling, but also helps in the shuttling of BMPs to the ventral side via the ECM of the Brachet’s cleft (Ben-Zvi et al. 2008; Plouhinec et al. 2013).

Studying **mammalian** embryogenesis is difficult due to the intrauterine development and thus lacking accessibility of the embryo. Therefore, little functional data on early mammalian dorsoventral patterning is available. Multiple BMP ligands are expressed during and are crucial for early mouse development, albeit partially redundant (Lyons et al. 1995; Solloway and Robertson 1999; Winnier et al. 1995). Chordin is expressed in the mouse organizer, the node, but is not required for the gross patterning of embryonic axes due to partial redundancy with the BMP inhibitor Noggin (Bachiller et al. 2000). Early in mouse development, BMP4 is expressed exclusively in extraembryonic tissue (Bachiller et al. 2000; Beck et al. 2002; Ben-Haim et al. 2006), and BMP4 signalling to the embryonic cells is important for primitive streak formation (reviewed in (Arnold and Robertson 2009)). With the embryo largely inaccessible, certain aspects of cellular communication and tissue patterning can be reconstructed in vitro using cell culture, gastruloids and blastoids. A synthetic system of BMP/Chordin secreting cells was shown to pattern a 2D culture, in agreement with BMP shuttling (Zhu et al. 2023). Furthermore, an increasing number of model systems derived from mouse or human embryonic stem cells (mESCs/hESCs) provide fascinating, developmentally-relevant insights. It was shown that colonies of hESCs can be patterned using BMP4 that is provided via the medium (Etoc et al. 2016). The resulting radial patterning within the colonies is the result of a reaction–diffusion system that requires Noggin. Especially gastruloids, 3D assemblies of ESCs that can partially recapitulate tissue differentiation and morphogenesis during development, will be useful systems to investigate mammalian DV patterning in the future. Mouse gastruloids, for example, break the initial symmetry and self-organize to form anterior–posterior as well as dorsoventral axes, without input from extraembryonic tissues (Beccari et al. 2018). The blastocyst-like blastoids, on the other hand, provide a valuable model system to study the communication between embryonic cells and extraembryonic trophectoderm (Rivron et al. 2018). In this context, it was shown that embryonically produced BMP4 signals to the trophectoderm, where it regulates proliferation and morphogenesis together with Nodal.

In summary, the central role of BMP2/4 and Chordin in the patterning of the DV axis is clearly ancestral for Bilateria. Mapping the orientation of the BMP signalling gradients in different bilaterian models on the phylogenetic tree tells us that the last common ancestor of Bilateria had to have a dorsal-to-ventral BMP signalling gradient—like in protostomes (Spiralia + Ecdysozoa) and non-chordate deuterostomes. A ventral BMP signalling maximum characteristic for cephalochordates and vertebrates suggests an axis flip at the base of Chordata, which made us, rather than the famous Geoffroy Saint-Hilaire’s lobster,^4^ “inverted” (Arendt and Nübler-Jung 1994; Geoffroy Saint-Hilaire 1822; Su et al. 2019). As bilaterians diversified, the BMP/Chordin-based patterning system underwent independent modifications sometimes involving the loss of Chordin. Moreover, in some clades, BMP signalling was “decommissioned” from DV patterning altogether and replaced by other mechanisms. Having established that, let us now address the situation with the BMP signalling outside Bilateria.

## BMP signalling in non-Bilateria

To-date, we know of four non-bilaterian phyla: Ctenophora (comb jellies), Porifera (sponges), Placozoa, and Cnidaria (corals and jellyfish). Whilst the phylogenetic positions of Ctenophora and Porifera have been a matter of intense debate during the last 15 years, all recent phylogenies retrieve Cnidaria as a robust bilaterian sister group, with Placozoa placed as a sister group to Bilateria + Cnidaria (Dunn et al. 2008; Feuda et al. 2017; Moroz et al. 2014; Pisani et al. 2015; Ryan et al. 2013; Schultz et al. 2023; Simion et al. 2017; Whelan et al. 2015) (Fig. 2). Ctenophores demonstrate a curious type of body symmetry: they are biradial. This means that they have a single oral-aboral (OA) body axis, and two axes of symmetry—one along their slit-like mouth, and another one perpendicular to it passing through the bases of their tentacles. Phylogenetic analyses of the available ctenophore sequences suggest that ctenophores do not seem to have Chordin and BMP orthologues, although several proteins clearly belong to the TGFβ superfamily of ligands (Genikhovich and Technau 2017; Pang et al. 2011).

Adult sponges are sessile and mostly lack a clear body axis (with the exception of asconoid and syconoid calcareous sponges, which are very clearly radially symmetric as adults), however, during their embryonic stages, all sponges display radial symmetry. The situation in sponges is intriguing: the genome of a homoscleromorph sponge *Oscarella* (Nichols et al. 2012) harbours a clear *Chordin* orthologue, however there is no statistical support for its several TGFβ molecules being *bona fide* BMPs. Other sequenced sponges do not seem to have either BMPs or Chordin (Kenny et al. 2020; Santini et al. 2023; Leininger et al. 2014).

Placozoans have been traditionally viewed as animals without any obvious symmetry and with the “dorsal” surface facing the water and “ventral” surface facing the substrate on which they crawl. Analyses by DuBuc and colleagues strongly suggest, however, that their “ventral” surface should be considered the oral, and their “dorsal” surface—the aboral end of their OA axis along which they became flattened (DuBuc et al. 2019). There is no trace of the second body axis either at the morphological or the molecular level. *Trichoplax* has a chordin-like molecule (DuBuc et al. 2019) grouping with vertebrate Kielin proteins, but no “real” Chordin, as well as several BMP molecules grouping with cnidarian and bilaterian BMPs albeit with low support.

**Fig. 3:**
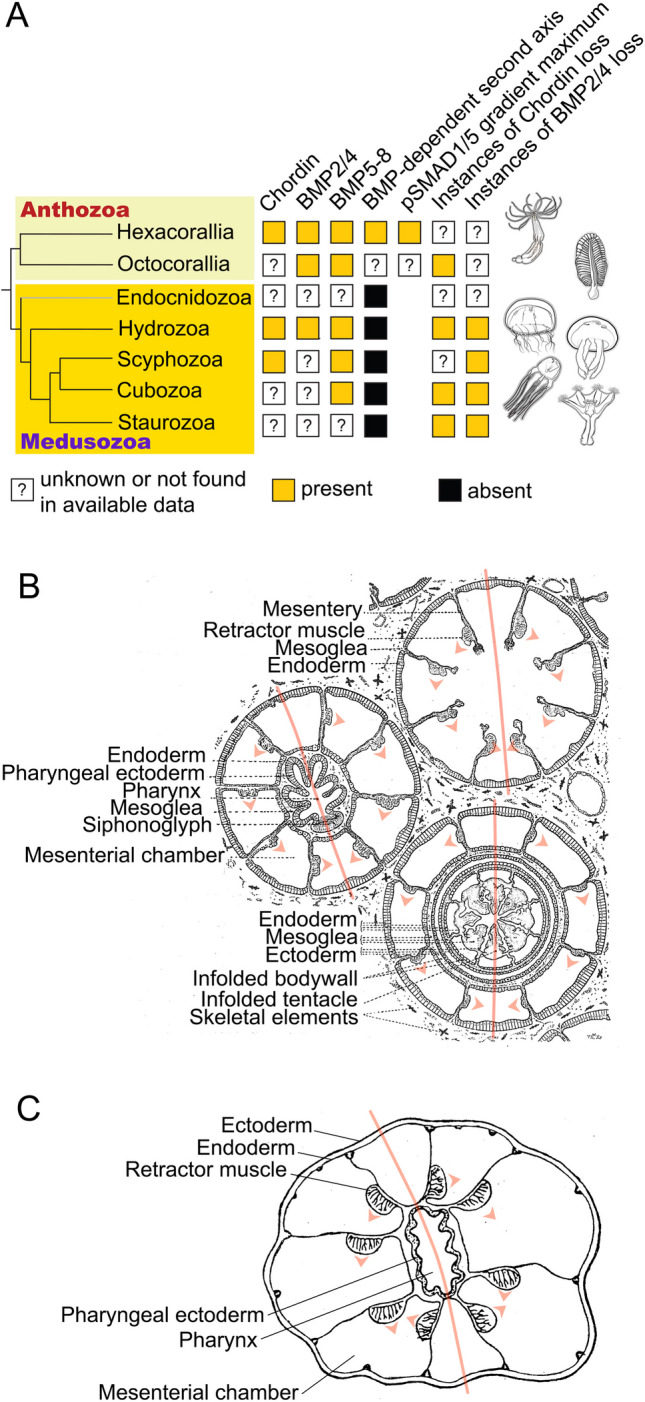
Cnidarian BMP signalling components and cnidarian bilaterality. **A** Cnidarian phylogeny and distribution of the key BMP-related traits in cnidarian classes. **B** Cross-section of the octocoral *Alcyonium digitatum* (dead man’s fingers). Modified from Kükenthal (1925). **C** Cross-section of the hexacoral *Halcampa duodecim-cirrata* (a twelve-tentacle burrowing sea anemone). Modified from Pax 1925. On **B** and **C**, orange lines show the orientation of the directive axis; arrowheads show the bilateral orientation of the retractor muscles on the mesenteries

Cnidaria (Fig. 3A), the bilaterian sister group, are divided into two clades: Medusozoa and Anthozoa (McFadden et al. 2021; Zapata et al. 2015). Medusozoa encompass the “conventional” cnidarian classes Hydrozoa, Staurozoa, Scyphozoa and Cubozoa, which we will address in some detail, as well as the parasitic Endocnidozoa, which were only recently recognized as cnidarians and will not be considered further in this review (Kayal et al. 2018). Anthozoa consist of Octocorallia (soft corals, sea pens etc.) and Hexacorallia (hard corals, sea anemones etc.) and are the slower evolving clade retaining many genes lost in medusozoans (Chapman et al. 2010; Gold et al. 2019; Hu et al. 2020; Khalturin et al. 2019; Leclère et al. 2019; McFadden et al. 2021; Putnam et al. 2007; Shinzato et al. 2011). Traditionally, cnidarians are described as radially symmetric, which is true for Medusozoa, but not for Anthozoa. The bilaterality of the anthozoan anatomy, which in some cases is secondarily replaced by biradiality, has been an accepted fact since at least the beginning of the twentieth century (Pax 1925; Stephenson 1928). In addition to the Wnt-dependent OA axis, which is a shared feature of all cnidarians, anthozoans have a second, “directive” axis perpendicular to the OA axis. At the level of morphology, bilateral symmetry manifests itself in the order of the formation of the gastrodermal folds called mesenteries, which subdivide the gastric cavity of anthozoans into mesenterial chambers sometimes referred to as segments, the position of the muscles on these mesenteries, and in the slit-like shape of the pharynx, which usually has a ciliated groove—the siphonoglyph—on one of its ends (Fig. 3B, C) (Berking 2007; Pax 1925; Stephenson 1928). All this did not prevent various textbooks from using sea anemones as examples of an animal with radial symmetry—they looked too much like a flower.

All intracellular components of BMP signalling (type I and type II BMP receptors, SMAD1/5, SMAD4) are present throughout Cnidaria, however, the situation is different when it comes to the secreted components of the BMP signalling, especially to the “core” BMP ligands BMP2/4 and BMP5/8, and their antagonists (Fig. 3A). Both, *Chordin* and *BMP2/4* orthologues are missing from the genomes of the staurozoans and cubozoans sequenced to-date (Kayal et al. 2018; Khalturin et al. 2019); the hydrozoans *Clytia*, *Hydractinia* and *Hydra* have lost *Chordin*, and have a TGFβ molecule, which may be a derived BMP2/4, whilst the scyphozoan *Aurelia* has a clear *Chordin* orthologue but no apparent *BMP2/4* has been published so far (Genikhovich and Technau 2017). Curiously, all medusozoans, which have been looked at, possess a *BMP5-8* orthologue, sometimes in several copies (Genikhovich and Technau 2017). *BMP5-8* and *BMP2/4* are expressed in specific radially symmetric domains along the oral-aboral (OA) axis in *Hydra* (Reinhardt et al. 2004; Watanabe et al. 2014), and there is an interesting report of a bilaterally symmetric expression of the putative *BMP2/4* and *BMP5*-8 at the aboral pole of the early planula larva in the hydroid *Podocoryne* (= *Hydractinia*) *carnea* (Reber-Müller et al. 2006); however, this result, in our opinion, is so unexpected and, potentially, so important that it needs to be independently confirmed.

In contrast to the medusozoans, anthozoans lost much fewer genes in the course of evolution (Fig. 3A). In the hexacorallian genomes we always find orthologues of *BMP2/4* and *Chordin* (as well as *BMP5-8*, *GDF5*, *ADMP*, *Gremlin*, *Noggin*, *Follistatin* etc.) (Putnam et al. 2007; Shinzato et al. 2011), however, the octocoral *Xenia* appears to lack a *Chordin* orthologue (Hu et al. 2020). Curiously, the most experimentally developed hexacoral model—the sea anemone *Nematostella vectensis*—lost one BMP inhibitor, Cerberus, which was retained in the hydrozoan *Clytia*. The wider appreciation of anthozoan bilaterality came after the publication of the expression pattern of *BMP2/4* on one side of the blastopore in a gastrula of the stony coral *Acropora millepora* soon followed by a similar observation in the sea anemone *Nematostella*: anthozoan embryos were clearly bilateral at the molecular level long before the first signs of morphological bilaterality became evident (Finnerty et al. 2004; Hayward et al. 2002; Matus et al. 2006a, b; Rentzsch et al. 2006).

**Fig. 4:**
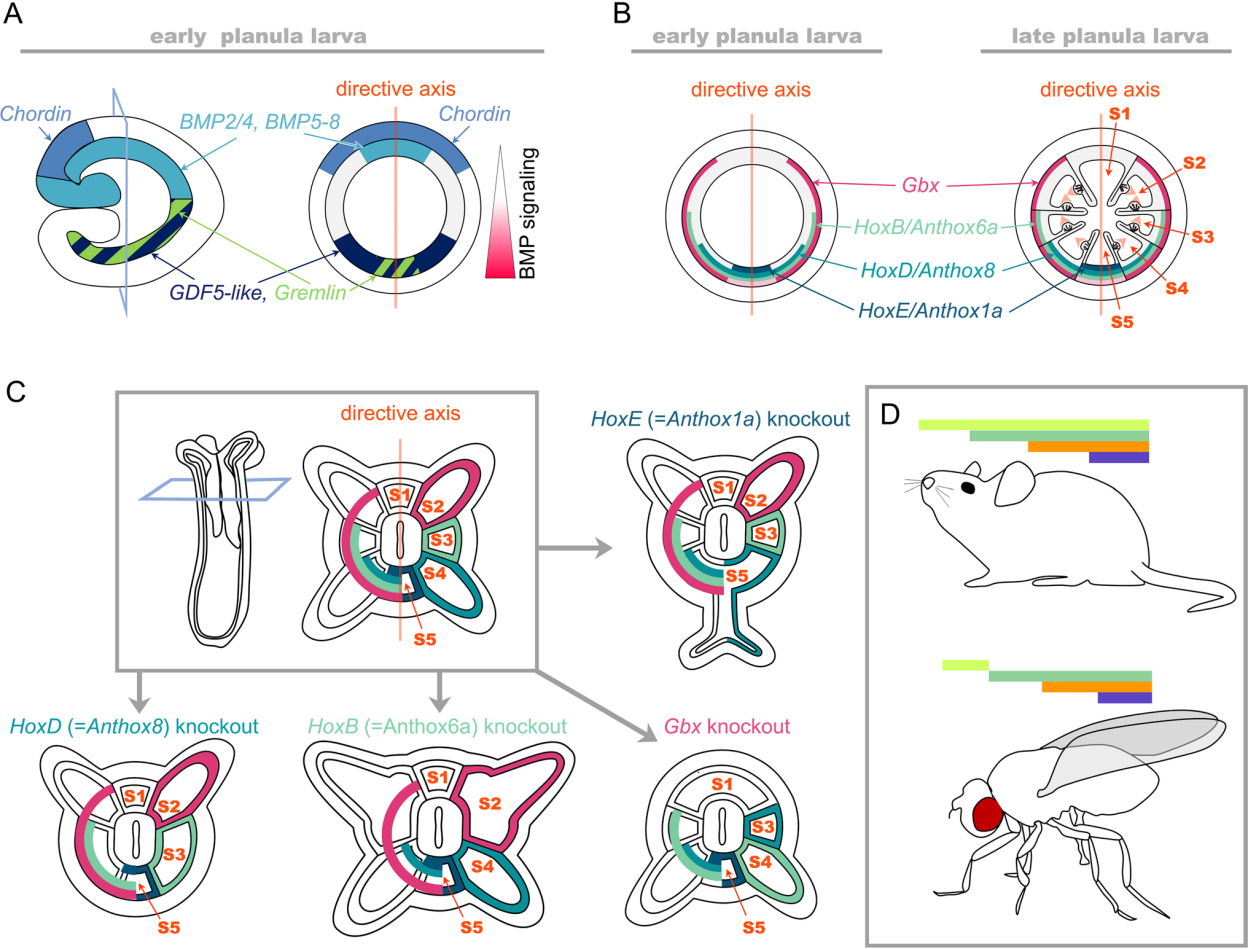
In *Nematostella*, BMP signalling controls directive axis patterning by regulating Hox genes. **A** Expression domains of the BMP ligands and BMP inhibitors in *Nematostella* early planula. **B** Expression of *Gbx* and *Hox* genes activated by BMP signalling in specific staggered domains along the directive axis of the Nematostella early planula and formation of the mesenteries at the boundaries of the *Gbx/Hox* expression. **C** In the primary polyp, each mesenterial segment acquires a unique *Gbx/Hox* code (shown on the left side of the cross-section). However, the fate of the segment is defined by one specific *Hox* gene or *Gbx* (shown on the right side of the cross-section) in a “posterior prevalence”-like manner. Knockouts of *Gbx* and Hox genes lead to specific homeotic transformations, where the mesenterial boundary disappears; the segment normally specified by the mutated *Gbx/Hox* gene fuses with and acquires the fate of its neighbouring segment located towards the *BMP2/4* side of the directive axis. Please note that paired segments are called S2, S3, and S4 here rather than S2/S8, S3/S7, and S4/S6 as in He et al. (2018). **D** Staggered Hox expression of the bilaterian Hox genes, which demonstrate a similar regulatory behaviour. Animal sketches on **D** were reproduced with permission from Technau and Genikhovich 2018

Thanks to the availability of the gene knockdown techniques we now know that, in *Nematostella*, the directive axis forms due to a BMP signalling-dependent symmetry break in the initially radially symmetric expression of the core BMP ligands BMP2/4 and BMP5/8, as well as of the BMP antagonist Chordin. This symmetry break establishes a gradient of BMP signalling along the newly formed directive axis, and the knockdown of any of these components leads to the loss of the gradient and complete radialisation of the embryo at the molecular and morphological level. Other components, such as the BMP ligand GDF5-like, the secreted BMP antagonist Gremlin, the putative BMP co-receptor RGM, the nuclear modulator of the pSMAD1/5 and potential transcriptional co-repressor ZSWIM4-6, and most certainly some other yet uncharacterized players, help shape the BMP signalling gradient and facilitate its robustness (Genikhovich et al. 2015; Knabl et al. 2024; Leclère and Rentzsch 2014; Saina et al. 2009). There are two notable features of the *Nematostella* BMP-dependent axis establishment and patterning: (i) *BMP2/4* and *BMP5-8* expression is negatively regulated by BMP signalling (Saina et al. 2009; Genikhovich et al. 2015; Knabl et al. 2024); (ii) at early stages, BMP signalling is highly dependent on Chordin. In *Nematostella*, *Chordin* is expressed on the same side of the directive axis as *BMP2/4* and *BMP5/8*, although their expression domains about each other (Fig. 4A). Antibody staining against nuclear pSMAD1/5 shows that the BMP signalling gradient forms at late gastrula stage and has its maximum on the *Chordin/BMP2/4/BMP5-8*-negative side of the directive axis, where BMP signalling activates the transcription of *GDF5-like* (another BMP) and of the BMP antagonist *Gremlin*, which help shape and maintain the signalling gradient (Fig. 4A). In late planula larva, *Chordin* expression stops, whilst *BMP* expression continues, however, the gradient of nuclear pSMAD1/5 along the directive axis disappears concomitantly with the shutdown of *Chordin* expression, and nuclear pSMAD1/5 starts to be observed in the same domain where the *BMP2/4* and *BMP5-8* genes are expressed (Knabl et al. 2024). *Chordin* and *BMP2/4* co-expression is very unusual but not unheard of in Bilateria. Similar to the situation in *Nematostella*, *Chordin* and *BMP2/4* are co-expressed on the ventral side of the embryo of the sea urchin *Paracentrotus lividus*, and ventrally produced Chordin is required for the establishment of the dorsal nuclear pSMAD1/5 maximum, which is suggestive of the Chordin-dependent BMP shuttling, just like in *Drosophila* or frog. However, like in other Bilateria, sea urchin Chordin knockdown results in the expansion of the BMP signalling domain (Lapraz et al. 2009, 2015), not so in the sea anemone. *Nematostella Chordin* knockdown results not in the upregulation of the BMP signalling but in its severe reduction and in the loss of the BMP signalling gradient in late gastrula/early planula (Genikhovich et al. 2015). Thus, it appears that *Nematostella* Chordin is not only responsible for the establishment of the pSMAD1/5 gradient with a maximum opposite to the Chordin source, but BMP signalling at the early stage is in principle Chordin-dependent. The current model is that *Nematostella* Chordin represses local BMP2/4/BMP5-8-mediated signalling thus preventing transcriptional repression of *BMP2/4* and *BMP5-8*. At the same time, Chordin shuttles BMP2/4/BMP5-8 heterodimers promoting BMP signalling (reinforced by the locally produced GDF5-like) at the opposite side of the directive axis. In the future, it will be interesting to understand the molecular mechanism of this apparent dependence of BMP signalling on Chordin at one developmental stage but not at another.

Taken together, we have two sister clades—Bilateria and Cnidaria—in which we find bilaterally symmetric animals with two orthogonal body axes patterned by graded Wnt (posterior-anterior and OA, respectively) and BMP signalling (DV and directive, respectively). Thus, it is possible that the last common ancestor of Cnidaria and Bilateria may have been bilaterally symmetric, that cnidarian and bilaterian body axes may be homologous, and that radially symmetric medusozoans may have lost their second body axis. Given the recurrent loss of *BMP2/4* or *Chordin* or both in different medusozoans it is tempting to speculate that this may be something more than a mere correlation. Should a radially symmetric medusozoan with a full set of “core BMPs” and Chordin be found, it would be interesting to explore whether or not these medusozoan BMPs and Chordin would be able to functionally substitute for their *Nematostella* counterparts, for which we know that they can generate bilaterality. However, there is a catch: we cannot rule out the possibility of convergent evolution of the BMP-dependent second axis in Bilateria and Anthozoa simply because BMP and Chordin represent a “symmetry breaking machine”. In one of his last papers, Hans Meinhardt analysed what was known about the regulatory relationships between Wnt and BMP in Cnidaria at the time and suggested that once the regulatory architecture was correct, it was “easy” to spontaneously create and maintain bilaterality (Meinhardt 2015). There is at least some experimental evidence in favour of this arguably even more exciting scenario.

## Potential convergence

**Fig. 5:**
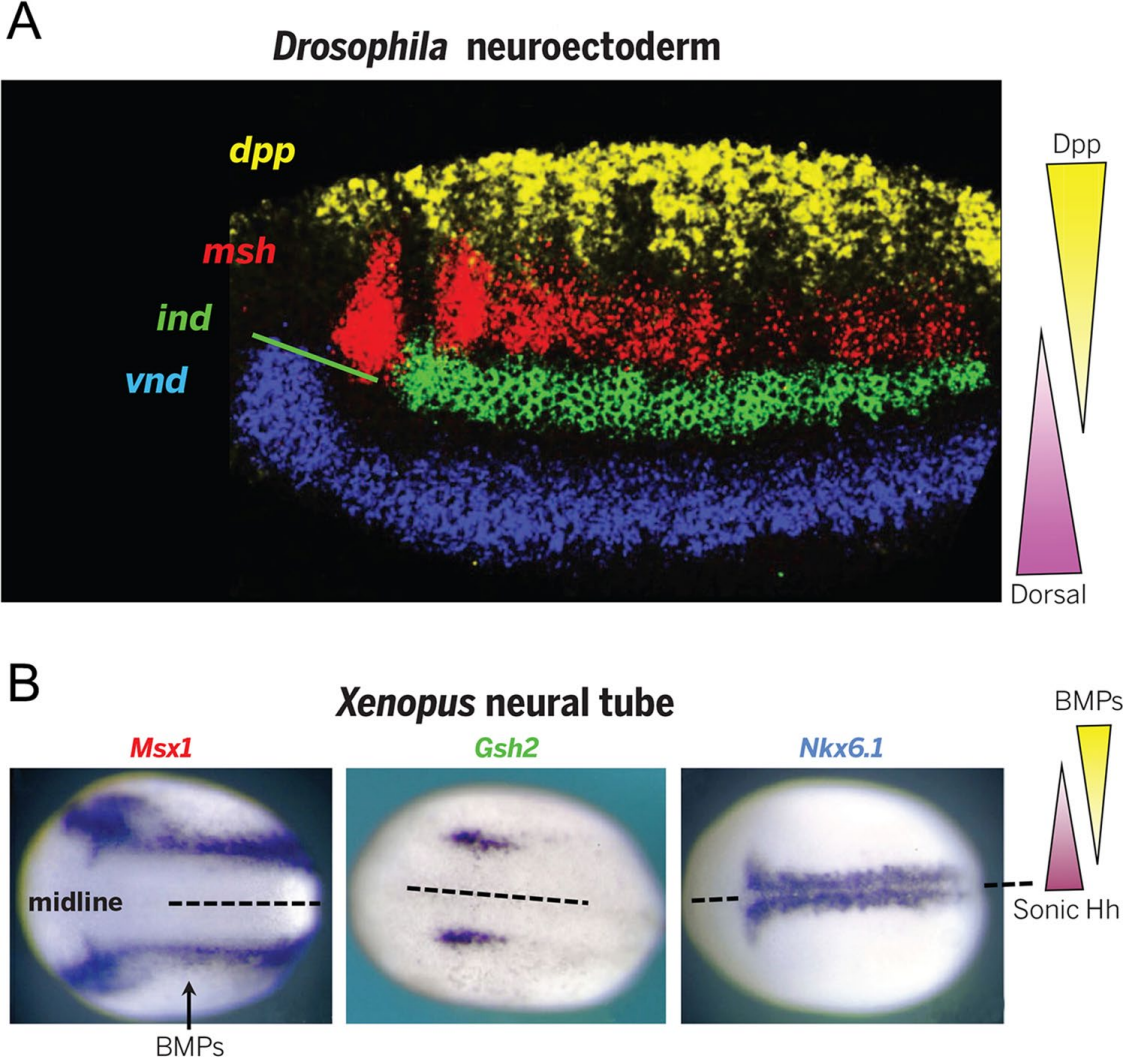
BMP signalling gradient regulates orthologous genes during neurectoderm patterning in the fly and in the frog. Reproduced with permission from Bier and De Robertis (2015)

We and others have shown that different intensities of Wnt/β-catenin signalling result in the activation of specific sets of genes in the oral “high Wnt” and in the aboral “low Wnt” areas of the *Nematostella* gastrula (Kraus et al. 2016; Lebedeva et al. 2021; Marlow et al. 2013; Röttinger et al. 2012). These sets of genes—*Brachyury*, *FoxA*, *FoxB*, and *Six3/6*, *FoxQ2*, *Fz5/8*—are canonical bilaterian posterior and anterior markers, respectively. Moreover, the regulatory logic of this patterning in *Nematostella* is like in deuterostomes; hence, we suggested that cnidarian OA axis is homologous to the bilaterian posterior-anterior axis (Lebedeva et al. 2021). The situation with the BMP-dependent axis is different. Part of global DV patterning in bilaterians with centralized nervous systems is the patterning of the neuroectoderm. In a classical example, BMP similarly affects neurectoderm patterning in *Drosophila* and *Xenopus*: in the dorsalmost area with the highest levels of BMP signalling, *Msh* is activated (*Msx1* in *Xenopus*; expressed in the ventralmost cells of the neural plate); in the intermediate levels of BMP signalling, *ind* is expressed (*Gsh2* in *Xenopus*); in the ventralmost neurectoderm with the lowest levels of BMP signalling, *vnd* is expressed (*Nkx6.1* in *Xenopus*; expressed in the cells of the dorsal midline) (Bier and De Robertis 2015; Fig. 5). Once activated in their respective domains, these transcription factors form a regulatory network based on mutual repressive interactions, which patterns the neurectoderm (Sagner and Briscoe 2019; Weiss et al. 1998). The orthologues of these genes exist in *Nematostella* (Zimmermann et al. 2023) but they do not seem to be controlled by BMP. Instead, graded BMP signalling is regulating axial patterning by directly activating a set of Hox genes and a non-Hox Antennapedia class homeobox gene *Gbx*. (Genikhovich et al. 2015; Knabl et al. 2024) (Fig. 4B). He et al. showed that their staggered expression is responsible for the formation of the mesenteries and providing each of the three paired (S2, S3, and S4) and two unpaired mesenterial chambers (S1 and S5) with unique molecular identities (He et al. 2018; Ryan et al. 2007; Fig. 4B, C). In this process, *Nematostella* Hox genes and *Gbx* display something suspiciously similar to the “posterior prevalence”, as we know it from vertebrates and arthropods. *HoxE/Anthox1a*, which is expressed in the unpaired mesenterial chamber (= segment) S5 at the former “high BMP signalling” end of the directive axis functionally overrides *HoxD/Anthox8* expressed in the segment pair S4 and S5. *HoxD/Anthox8* functionally overrides *HoxB/Anthox6a* expressed in the segments S3, S4, and S5. Finally, *HoxB/Anthox6a* functionally overrides *Gbx* expressed in the segments S2, S3, S4, and S5 (Fig. 4C). The mechanism of this “prevalence” is unknown and needs to be experimentally addressed. The obvious difference to the bilaterian Hox genes is that, in *Nematostella*, Hox genes are involved not only in conveying axial identities to segments, but in the formation of the segment boundaries. Staggered expression of the *Nematostella* Hox genes along the directive axis was so suggestive that it revived the nineteenth century idea that anthozoan directive axis may be homologous to the bilaterian anterior–posterior axis (Fig. 4C, D) (Arendt et al. 2016; Jägersten 1955; Nielsen et al. 2018; Remane 1950; Sedgwick 1884; van Beneden 1891). This remains a matter of debate, given that cnidarian OA axis shares many regulatory features with the bilaterian posterior-anterior axis, that bilaterian Hox genes are expressed along the posterior-anterior body axis, which is controlled by Wnt rather than by BMP, and that cnidarian Hox genes are most likely not direct orthologues of the bilaterian Hox genes but rather a product of the Cnidaria-specific diversification of the Hox cluster of the cnidarian-bilaterian ancestor, which likely contained a single “anterior” and a single “non-anterior” Hox gene (reviewed in more detail in Genikhovich and Technau 2017). Possibly, however, these ancestral Hox genes were already expressed in staggered domains and capable of overriding each other’s function.

## Conclusion

With this review, we cannot answer the question whether the last common ancestor of Cnidaria and Bilateria was bilaterally or radially symmetric or whether the emergence of the Chordin-mediated BMP shuttling was the evolutionary event, which made bilaterally symmetric body plans possible. However, we hope that we managed to convey a different message: BMP signalling-dependent patterning of the secondary body axis in animals is at the same time very conserved and very evolvable. Often, superficial differences originate from a detectable ancestral pattern, whilst superficial similarities may arise convergently based on the intrinsic properties of the BMP-Chordin system to break symmetry and activate downstream transcription factors in a concentration-dependent manner.

## Data Availability

No datasets were generated or analysed during the current study.

## Footnotes

1 Although vertebrates also have Smad8 and Smad9, we will to refer to all the Smad1/5/8/9 homologs as Smad1/5 in this article.

2 In this review, we will mention *Drosophila* gene and protein names when we encounter them for the first time, but we will then keep using their vertebrate orthologue names even when talking about *Drosophila* genes and proteins to keep the terminology consistent.

3 Phylogenetic analyses showed that xenacoelomorphs *Xenoturbella bocki* and *Symsagittifera roscoffensis* have two ADMP genes, which are orthologous to the *ADMP1* and *ADMP2* of ambulacrarians (Chang et al. 2016). This can be seen either as additional evidence in favour of the phylogenetic position of Xenacoelomorpha as a sister to Ambulacraria or indicate multiple independent losses of *ADMP2* across Bilateria.

4 Étienne Geoffroy Saint-Hilaire (1772–1844)—a French naturalist who noticed that the relative positions of the central nervous system, heart and gut in a lobster were the same as in a mammal but the ventral most lobster structure corresponded to the dorsal most mammalian structure, hence the name for the “inverted lobster hypothesis”.
